# Probiotics-Supplemented Low-Protein Diet for Microbiota Modulation in Patients with Advanced Chronic Kidney Disease (ProLowCKD): Results from a Placebo-Controlled Randomized Trial

**DOI:** 10.3390/nu14081637

**Published:** 2022-04-14

**Authors:** Andreana De Mauri, Deborah Carrera, Marco Bagnati, Roberta Rolla, Matteo Vidali, Doriana Chiarinotti, Marco Pane, Angela Amoruso, Mario Del Piano

**Affiliations:** 1 Nephrology and Dialysis Unit, Maggiore della Carità University Hospital, 28100 Novara, Italy; doriana.chiarinotti@maggioreosp.novara.it; 2 Dietetic and Clinical Nutrition, Maggiore della Carità University Hospital, 28100 Novara, Italy; deborah.carrera@libero.it; 3 Clinical Chemistry Laboratory, Maggiore della Carità University Hospital, 28100 Novara, Italy; marco.bagnati@maggioreosp.novara.it (M.B.); roberta.rolla@med.uniupo.it (R.R.); 4 Department of Health Sciences, Amedeo Avogadro University of Eastern Piedmont, 28100 Novara, Italy; 5 Clinical Chemistry Unit, Fondazione IRCCS Ca’ Granda Maggiore Policlinico Hospital, 20122 Milano, Italy; matteo.vidali@gmail.com; 6 Research & Development, Probiotical Research Srl, 28100 Novara, Italy; m.pane@mofinalce.it (M.P.); a.amoruso@mofinalce.it (A.A.); 7 Past head of Clinical Research, Probiotical SpA, 28100 Novara, Italy; mariodelpiano3@gmail.com

**Keywords:** low protein diet, chronic kidney disease, gut microbiota, microbial uremic toxins, probiotics, ecologic therapy, green nephrology

## Abstract

The probiotics-supplemented low-protein diet in chronic kidney disease (ProLowCKD) was a single-centre, double-blind, placebo-controlled, randomised trial that was conducted to investigate whether the association between a low protein diet (LPD) and a new formulation of probiotics (*Bifidobacterium longum* and *Lactobacillus reuteri*) was effective at reducing traditional uremic, microbiota-derived, and proatherogenic toxins in sixty patients affected by advanced CKD. After 2 months of a LPD—a reduction in blood urea nitrogen (52 ± 17 vs. 46 ± 15 mg/dL, *p* = 0.003), total cholesterol (185 ± 41 vs. 171 ± 34 mg/dL, *p* = 0.001), and triglycerides (194 ± 148 vs. 161 ± 70 mg/dL, *p* = 0.03) was observed; 57 subjects were then randomized to receive probiotics or a placebo for the subsequent 3 months. A total of 27 patients in the placebo group showed increased serum values of total cholesterol (169 ± 36 vs. 185 ± 40 mg/dL, *p* = 0.01), LDL cholesterol (169 ± 36 vs. 185 ± 40 mg/dL, *p* = 0.02), lipoprotein-associated phospholipase A_2_ (155.4 ± 39.3 vs. 167.5 ± 51.4 nmol/mL/min, *p* = 0.006), and indoxyl-sulphate (30.1 ± 17.6 vs. 34.5 ± 20.2 μM, *p* = 0.026), while the 24 subjects in the probiotics group showed a trend in the reduction of microbiota toxins. A reduction of antihypertensive and diuretic medications was possible in the probiotics group. This study shows that associating probiotics to LPD may have an additional beneficial effect on the control and modulation of microbiota-derived and proatherogenic toxins in CKD patients.

## 1. Introduction

Chronic kidney disease (CKD) is characterized by the accumulation of a number of metabolites—referred to as uremic toxins—that cannot be excreted by failing kidneys [1]. Traditional uremic toxins are risk factors for cardiovascular diseases (CVD) [2]. Other non-traditional risk factors for CVD include gut-derived toxins produced by the intestinal microbiota.

The term “microbiota” describes the populations of bacteria, fungi, and viruses that symbiotically live in a host [3,4], contributing to morphogenesis and homeostasis [5,6]. While an eubiotic gut microbiota is characterized by a saccharolytic metabolism, consisting of the fermentation of carbohydrates and undigested oligosaccharides (prebiotics) into beneficial short-chain fatty acids, a dysbiotic microbiota has a proteolytic metabolism, generating branched-chain fatty acids, ammonia, amines, indoles, phenols, and other toxic agents [7,8].

Dysbiotic microbiota is a feature of patients affected by CKD, as urea promotes the overgrowth of proteolytic bacteria (actinobacteria, proteobacteria, and firmicutes) [9,10], increases the permeability of the intestinal wall, and induces the translocation of bacteria or their fractions into the bloodstream, enhancing accelerated atherosclerosis and systemic inflammation [11].

P-resyl-sulphate (PCS), derived from tyrosine and phenylalanine phenolic metabolites after liver sulphation, and indoxyl-sulphate (IS), derived from tryptophan indole metabolites after liver sulphation, are the most studied microbial toxins. They are correlated with the progression of renal failure and cardiovascular morbidity and mortality in CKD [12,13,14].

In addition to the traditional and microbiota-derived uremic toxins, several new predictors of cardiovascular events have recently been recognized, such as lipoprotein-associated phospholipase A_2_ (Lp-PLA_2_). Lp-PLA_2_, is a serine lipase produced by activated monocytes, which induces the chemotaxis of leucocytes into the lipid core of the atherosclerotic plaque, transforming it into a necrotic and instable core [15]. As a matter of fact, consequence, Lp-PLA_2_ is recognized to predict acute cardiovascular events [16,17].

In this context, it is pivotal to reduce the burden of traditional uremic and microbiota-related toxins in CKD patients, and the nutritional approach represents a major intervention [18].

The milestone of nutritional therapy in CKD is the low protein diet (LPD), consisting of a protein intake of 0.6-g/kg of body weight or less, is energy and vegetable-enriched, and sodium and phosphorus-depleted. LPD reduces uremic symptoms, increases the dialysis-free time [19,20], is safe, and low-cost. Recent studies suggest that the role of LPD in reducing uremic toxins is enhanced by associating prebiotics and probiotics [21,22].

While prebiotics are “nondigestible food ingredients (mainly found in vegetables) that beneficially affect the host by selectively stimulating the growth and/or activity of bacterial species that improve host health” [23], probiotics, in 1989, were defined as “live microbial feed supplement which beneficially affects the host” [24]. Probiotics alone failed to reshape the microbiota in CKD, while a combination of probiotics, mainly Lactobacillus or *Bifidobacterium* spp., and prebiotics, mainly fructo-oligosaccharides (FOS), successfully reduced the uremic toxin overload [25,26,27,28].

Despite many studies with controversial results, literature still lacks clinical trials evaluating the synergic effects of LPD and selected probiotics in modulating gut microbiota, restoring safer metabolic profiles, and preserving residual renal function in subjects with advanced renal disease.

The aim of the probiotics-supplemented low-protein diet in chronic kidney disease (ProLowCKD) protocol was to evaluate whether the association of selected probiotics on top of a low protein diet was able to reduce the burden of uremic, microbiota-derived, and proatherogenic toxins in patients with advanced renal failure who were not on dialysis.

## 2. Methods

The probiotics-supplemented low-protein diet in chronic kidney disease (ProLowCKD) was a single-centre, double-blind, placebo-controlled, randomized study. A detailed description of the ProLowCKD study protocol has been published elsewhere [29].

The ProLowCKD trial was registered on ClinicalTrials.gov (number NCT04204005, access on 18 December 2019).

The study was conducted in accordance with the Declaration of Helsinki (2000) of the World Medical Association. The study was approved by the Ethical Committee of our institution (215/CE n. CE 3/17) on 13 March 2017. Every patient enrolled signed an informed consent.

### 2.1. Participants

Patients aged 18 to 80 years, affected by CKD, with eGFR less than 25 mL/min/1.73 m^2^, and who were not on dialysis, were eligible. Subjects with previous renal transplantations, chronic inflammatory bowel disease, who refused to sign the informed consent, or refused LPD, were excluded; subjects receiving antibacterial therapy or initiating dialysis during the study period were excluded.

### 2.2. Intervention

Low protein diet composition: according to the most recent recommendations [18], 0.6 g of protein/kg of body weight/day, 50% of high biological value, 25–30 kcal/kg/day of energy intake, less than 6 g/day of salt, less than 800 mg/day of phosphorus, less than 10% of monounsaturated fats, less than 300 mg/day of cholesterol, and a high fibre content (more than 7 g/1000 kcal). Free-protein products were prescribed, if necessary.

Vitamin B12, folic acid, calcium, vitamin D, iron, and erythropoietin supplementations were prescribed, when necessary.

Adherence to the diet was tested through the total urine nitrogen (TUN) excretion according [30] to the Maroni–Mitch formula: TUN = urine urea (g/day) + 0.031 * body weight, the protein catabolic rate (PCR) according to the formula PCR = 6.25 * TUN (g/day), and normalized PCR (nPCR), according to the formula PCR/body weight.

Prebiotics were provided, together with the LPD, in light of its high content of plant-based foods and free protein products.

Probiotics composition: 5 × 10^9^ of Bifidobacterium longum (mix DLBL), 1 × 10^9^ Lactobacillus reuteri LRE02 (DSM 23878) and maltodextrin (total 2 g). The probiotic species employed were granted the Qualified Presumption of Safety (QPS) status by the European Food Safety Authority (EFSA) in 2007. Probiotics were provided by Probiotical S.p.A. (Novara, Italy).

Placebo: maltodextrin (2 g).

### 2.3. Study Design

At enrolment time (T0), the protocol was illustrated, and the informed consent collected. Participants were prescribed LPD in addition to their ongoing pharmacological therapy; after 2 months (T2), they were randomized in a 1:1 ratio to receive, in addition to the LPD, either probiotics (probiotics group) or a placebo (placebo group), according to the registration order at the enrolment visit. They were invited to assume two doses per day of probiotics (or placebo) for 1 month and one dose for the following 2 months.

At T0, T2, and T5 routine laboratory measurements (haemoglobin, urea, creatinine, mean urea, and creatinine clearance, estimated GFR (eGFR), according to the CKD-EPI equation, sodium, potassium, uric acid, calcium, phosphate, PTH, acid-base balance, CRP, albumin) were measured on an ADVIA^®^ 1800 Clinical Chemistry Analyzer (Siemens Healthcare Diagnostics, Marburg, Germany). Total and free serum p-cresyl sulphate (t- and f-PCS) and total and free serum indoxyl sulphate (t- and f-IS) were measured through a high-performance liquid chromatography technique coupled with tandem mass spectrometry (B.S.N. Srl, Castelleone (CR) Italy). Serum Lp-PLA_2_ activity was measured through the new PLAC^®^ test (Diazyme Laboratories, Inc. 12889 Gregg Court, Poway, CA 92026 USA).

Quality of life was tested by the Short Form-36 (SF-36), which was validated in CKD subjects [31,32].

Nutritional assessment was evaluated through the physical exam: body weight, height, BMI (kg/m^2^), middle upper arm circumference (cm), triceps skinfold (mm) of the left arm using the skinfold caliper “Holtain Tanner” (Holtain, Ltd., Crymych, UK), dominant hand grip strength (kg) using Hydraulic Hand Dynamometer Owner’s Manual (Sammons Preston), according to the references [33,34]. Bioelectrical impedance analysis (BIA) on the Akern model 101 tool (Akern Srl, Pisa, Italy) was performed to estimate total body water (TBW), fat mass (kg), fat-free body mass (kg), and phase angle.

Follow-up: patients were followed-up with for three years and acute cardiovascular events, such as cardiac (ischemia and congestive heart failure), cerebral (stroke or acute transient ischemia), and peripheral vascular (lower limb ischemia) accidents, as well as progression to end stage kidney disease and dialysis start, were recorded.

### 2.4. Statistical Analysis

A statistical analysis was performed on the SPSS statistical software v.17.0 (SPSS Inc., Chicago, IL, USA). Normality distribution was preliminarily tested by Shapiro–Wilk and q–q plot tests. Quantitative variables were expressed as median (minimum–maximum) or mean (±standard deviation) for not normally and normally distributed continuous variables, respectively. Qualitative variables were expressed as absolute and relative frequencies. Differences between groups for continuous variables were estimated by the nonparametric Mann–Whitney U-test (for independent samples) and the Wilcoxon signed rank test (for paired samples) or by the parametric paired T-test. Disease-free survival was evaluated by the Kaplan–Meier analysis, while differences between groups were studied by log rank and the Breslow test. A *p* < 0.05 was considered statistically significant.

## 3. Results

**Figure 1:**
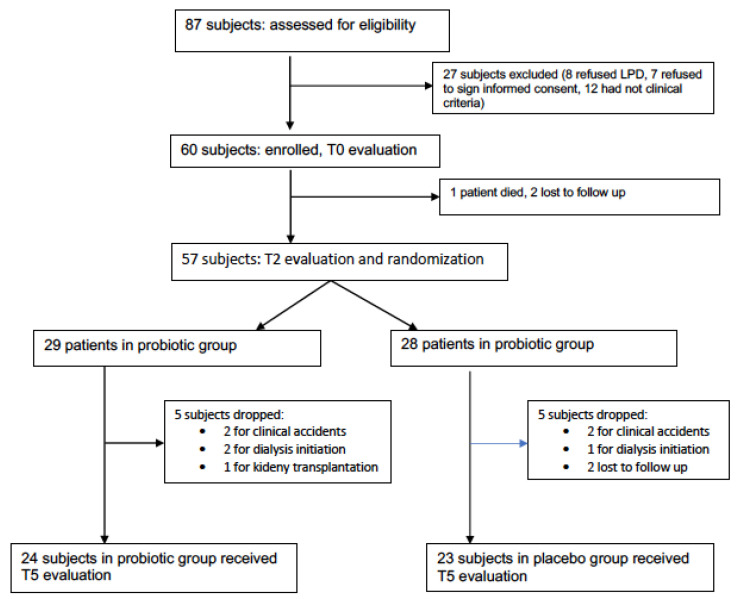
Flow chart of patient evaluations.

A total of 87 subjects were investigated for eligibility: 27 were excluded (12 did not satisfy the enrolment criteria, 8 refused nutritional therapy, and 7 refused to sign the informed consent). Sixty patients were enrolled, aged 64.8 ± 11.8 years, 70% males, 88% with hypertension, 28% with diabetes, and 16% with coronary artery disease. A total of 57 subjects received the T2-evaluation (1 patient died and 2 were lost to follow-up) and 47 completed the T5 evaluation (10 subjects dropped out: 1 for renal transplantation, 3 for dialysis initiation, 4 for clinical events, and 2 were lost to follow-up) (Figure 1). Characteristics of the enrolled subjects are listed in Table 1.

After 2 months of run-in periods with LPD, a significant reduction in BUN (52 ± 17 vs. 46 ± 15 mg/dL, *p* = 0.003), total cholesterol (185 ± 41 vs. 171 ± 34 mg/dL, *p* = 0.001), and triglycerides (194 ± 148 vs. 161 ± 70 mg/dL, *p* = 0.03) were observed, without any difference in respect to other biochemical and physical parameters. TUN (10.8 ± 3.4 vs. 9.3 ± 3.1 g/kg/day, *p* = 0.0006), protein catabolic rate (67.5 ± 2.1 vs. 58.4 ± 19.4 g/day, *p* = 0.0001), and nPCR (0.90 ± 0.30 vs. 0.77 ± 0.2, *p* = 0.007) significantly decreased, according to the compliance to the diet (Table 2).

### 3.1. Changes in Uremic Toxins

At T5, in comparison with T2, the placebo group showed increased values of Lp-PLA_2_ (155.4 ± 39.3 vs. 167.5 ± 51.4 nmol/mL/min, *p* = 0.006) and t-IS (30.1 ± 17.6 vs. 34.5 ± 20.2 μM, *p* = 0.026), while subjects receiving probiotics showed a trend in reduction of microbiota-derived toxins, even if not statistically significant (Table 3). No differences between the probiotics group and the placebo group were detected at T2.

### 3.2. Changes in Biochemical Parameters and Drug Therapy

Despite the absence of any significant difference in the glomerular filtration rate, the probiotics group showed lower daily urine protein excretion (1.2 ± 1.6 vs. 1.9 ± 2.1 g/24 h in the placebo group, *p* = 0.03), which remained stable during the follow-up, while protein excretion significantly increased in the placebo group (1.9 ± 2.1 g/day at T2 to 2.7 ± 2.4 g/day at T5, *p* = 0.04). During the follow-up, total and LDL cholesterol significantly increased in the placebo group (169 ± 36 mg/dL vs. 185 ± 40, *p* = 0.01 and 90 ± 28 vs. 104 ± 35 mg/dL, *p* = 0.04, respectively), while a stability of these parameters was observed in the treated group (Table 4). The adherence to the diet remained stable, as demonstrated by the values of TUN, PCR, and nPCR in both groups. No differences between the probiotics group and the placebo group were detected at T2, with regard to the remaining parameters.

In addition, an amelioration of blood pressure levels and signs of expansion of extracellular fluid volume (e.g., oedema) were observed in the probiotics group, allowing a significant reduction of the dose of antihypertensive medications (1.6 ± 1.1 vs. 1.3 ± 0.9 doses per day, *p* = 0.04) and loop diuretics (33.7 ± 51.4 vs. 14.6 ± 25.4 mg per day, *p* = 0.008) (Table 5).

### 3.3. Changes in Nutritional Status and Quality of Life

Good nutritional status with no variations during the follow-up was observed in both groups (Table 6); in contrast, in the probiotics group, we observed a statistically significant improvement in the emotional functioning item of the Sf-36 questionnaire (Table 6). No differences between the probiotics and placebo group were detected at T2.

### 3.4. Survival Analysis

**Figure 2:**
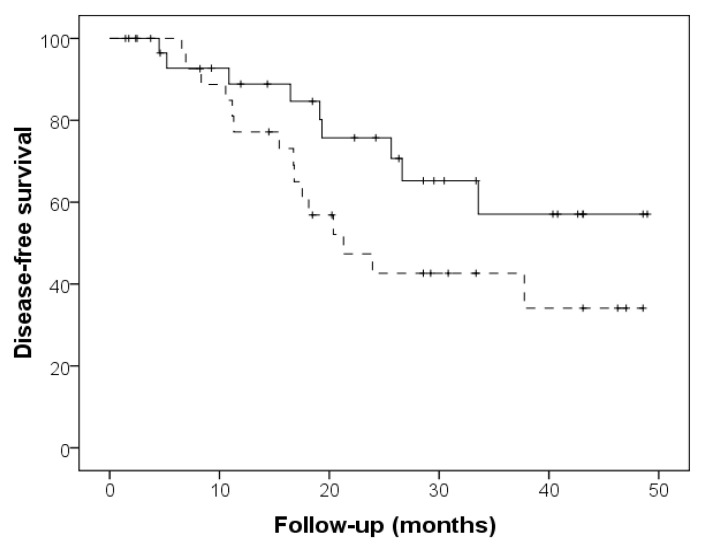
Dialysis-free survival in the probiotics group (continuous line) and placebo group (dashed line) (log rank *p* = 0.080).

**Figure 3:**
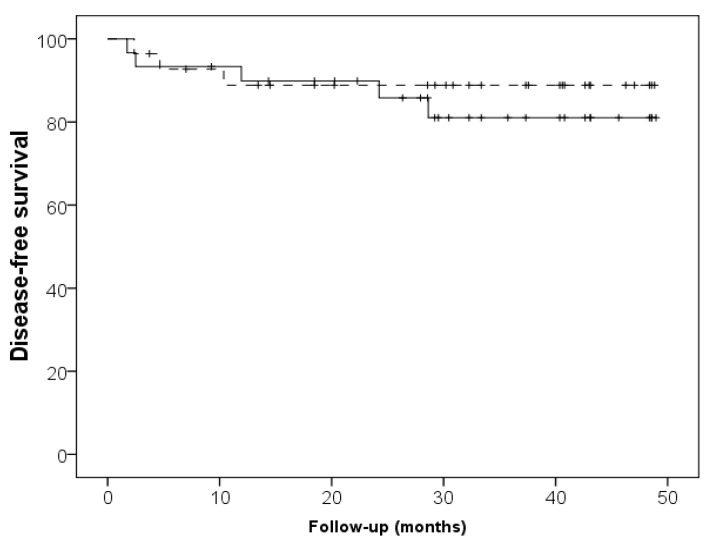
Cardiovascular event-free survival in the probiotics group (continuous line) and placebo group (dashed line) (log rank *p* = 0.5).

Mean follow-up for enrolled subjects was 31 ± 14 months. Interestingly, patients assuming probiotics displayed a trend in reduction of the progression to end stage renal disease and dialysis initiation, in comparison with subjects assuming placebo (cumulative survival 57% vs. 34%, log rank *p* = 0.08, (Figure 2). No differences between the two groups were, however, observed in respect to the incidence of cardiovascular events (cumulative survival 81% vs. 89%, log rank *p* = 0.55) (Figure 3).

## 4. Discussion

The overall results of this study seem to demonstrate that a probiotics-supplemented low protein diet prevents the increase of serum levels of IS, Lp-PLA_2_ activity, serum total cholesterol and, marginally, of PC, as well as allows the reduction of loop diuretics and antihypertensive agent use, improves quality of life and, finally, delays the progression to end stage renal disease and the need for dialysis.

In this context, the combination of LPD and probiotics could be named “microbiota modulating therapy” and our intervention is focused on the three “P’s” (protein, prebiotics, and probiotics). The milestone of the nutritional intervention in CKD patients remains the LPD [20], which acts on the gut microbiota through two mechanisms: first, the reduction in protein intake decreases the substrates for the proteolytic bacteria; second, the high content of soluble and insoluble fibres ensure an appropriate intake of prebiotics (second P) and promote the saccharolytic metabolism [35,36]. In fact, Black et al. [21] reported reduced levels of PCS in CKD patients treated with LPD for 6 months, and Marzocco [22] observed reduced levels of IS in CKD patients treated with a very low protein diet for only 1 week. In our population, we observed a trend in reduction of PCS and Lp-PLA_2_ before and after the randomization time, supporting these observations. In addition, the protein-free flours, often added to the diet regimen, are enriched with fibres, fructo-oligosaccharides, inulin, and other prebiotics that ameliorate the properties of baked products.

Enrolled subjects were already followed in our outpatient service and had previous nephrological counselling in earlier stages of renal disease, with state-of-the-art treatment, as showed by optimal control of biochemical parameters, nutritional status, and bioimpedance parameters [37] at baseline: consequently, the optimal baseline condition could have weakened the beneficial effects of subsequent interventions.

As uremic microbiota is characterized by a significant increase in Actinobacteria, Proteobacteria, and a reduction in *Bacteriaceae* and *Lactobacillaceae* families [4,5], it is not surprising that interventional trials focus on *Lactobacillus* and *Bifidobacteria* to restore the saccharolytic colonic flora. The most frequently administered probiotics are *L. acidophilus*, *L. casei*, *L. sakei*, *L. reuteri, Bifidobacterium longum*, *B. bifidum*, and *Streptococcus thermophilus* [35,38,39], with either negative, positive, or neutral correlations between probiotic supplementation and uremic toxins. This trial was conducted using two strains of bacteria with high anti-inflammatory properties and high in-vitro survival and replication rate (*Bifidobacterium longum*, mix DLBL, and *Lactobacillus reuteri* LRE02, DSM 23878).

Interestingly, in our study, in accordance with previous papers [40,41], prebiotics prevented the increase of IS levels. As indole is usually produced by colonic bacteria expressing tryptophanase, probiotics could act either through a direct/indirect “competitive” mechanism of inhibition/modulation of these bacteria. Otherwise, probiotics could restore the intestinal wall permeability, reducing indole absorption into the bloodstream.

In the present trial, one more effect of probiotics involved preventing the increase of the Lp-PLA_2_ serum level. As previous studies revealed that plant-based diets reduced the serum lipid profile in the general population [42,43], Lp-PLA_2_ included [44], our work is the first trial, to our knowledge, that demonstrates a possible relationship between probiotics-supplemented LPD and Lp-PLA_2_ in subjects with advanced CKD. Since no studies unfold a possible link between Lp-PLA_2_ and any bacterial metabolism, we would argue that either some unknown metabolic pathway, or the reduction of LDL cholesterol in which Lp-PLA_2_ is linked, induce the observed decreased levels of Lp-PLA_2_ In fact, in the placebo group, an elevation of total cholesterol and LDL cholesterol was observed, maybe due to a primary impairment in the urine protein excretion until the nephrotic range, while in the placebo group, the urine proteins were lower and remained stable.

One more interesting finding of our study is the reduction of the dose of antihypertensive drugs and loop diuretics observed in the probiotics group, due to an amelioration of blood pressure and extracellular fluid volume balance. This finding confirms a general improvement in the clinical status with important health perception impacts, as demonstrated by the increased emotional role functioning in the probiotics group.

Finally, we found that probiotic supplementation was associated with longer dialysis-free survival, even if the survival analysis did not reach the statistically significance: 17% of patients in the probiotics group needed renal replacement therapy, compared to 50% in the placebo group. No differences between groups were found in the incidence of cardiovascular events, perhaps due to the low number of events observed during the study period.

The main limitation of this study was the low number of enrolled subjects and the short treatment time (only three months of probiotics/placebo); this could explain the fact that probiotics supplementation seemed to prevent worsening rather than allow improvement of the basal condition, particularly regarding microbiota-derived uremic toxins.

However, our study presents some strengths: first, the holistic approach, focused on the biochemical and biophysical parameters, quality of life, pharmacological therapy, and long follow-up; second, we showed that the association of LPD with probiotics is safe because no adverse effects were observed, and no malnutrition was detected. Third, as previously stated, for LPD alone [45,46], probiotics-supplemented LPD is a low-cost therapy, with advantages, i.e., via a reduction in dialysis need and in pharmacological overload.

Finally, a probiotics-supplemented LPD may have a role in the “green nephrology approach”, as illustrated by Piccoli et al. [47], through several mechanisms, such as a tapering of waste products in pharmaceutical industries (drugs and dialysis) as well as more favourable ecologic impacts, due to a reduced use of animal-derived products in the diet.

## 5. Conclusions

Our study demonstrates that, in advanced CKD, the “3P’s” approach with a probiotics-supplemented low protein diet reduces traditional uremic, microbiota-derived and atherogenic uremic toxins, seems to delay the progression to end stage renal disease, reduces drug overload, and improves one’s quality of life without increasing the incidence of adverse effects or malnutrition. It could represent a good option to modulate microbiota in CKD patients.

We believe that a probiotics-supplemented low protein diet represents not only a holistic kidney-friendly approach but allows for ecological and planet-friendly management of renal disease, which will have an increasing role in the near future.

## Figures and Tables

**Table 1:** Characteristics of the enrolled subjects.

Total patients	N. 60
Age (years, mean ± SD)	64.8 ± 11.8
Male/female (N/%)	42 (70%)
Hypertension N (%)	53 (88%)
Diabetes N (%)	17 (28%)
Coronary artery disease N (%)	10 (16%)
Cause of CKD	
Hypertension N (%)	24 (40%)
Diabetes N (%)	9 (15%)
Genetics (N%)	12 (20%)
Others N (%)	15 (25%)

**Table 2:** Comparison between biochemical parameters and pharmacological therapy in all patients. Values are presented as mean ± SD.

	T0	T2
Lp-PLA_2_ (nmol/mL/min)	164.8 ± 43.9	160.6 ± 51.4
t-PC (μM)	135.3 ± 78.4	120.7 ± 69.9
f-PC (μM)	5.21 ± 3.89	4.1 ± 3.1
t-IS (μM)	30.5 ± 14.6	30.2 ± 20.2
f-IS (μM)	1.44 ± 0.82	1.35 ± 0.99
EPI-CKD (mL/min)	18.1 ± 3.6	18.2 ± 3.7
Urine proteins (g/24 h)	1.6 ± 1.4	1.7 ± 1.9
Hb (g/dL)	12.0 ± 1.5	11.9 ± 1.5
**BUN (mg/dL)**	**52 ± 17**	**46 ± 15 ^1^**
Uric acid (mg/dL)	6 ± 1.5	6 ± 1.2
Albumin (mg/dL)	4.2 ±0.3	4.1 ± 0.3
Calcium (mg/dL)	9.1 ± 0.6	9.1 ± 0.5
Phosphorus (mg/dL)	3.7 ± 0.7	3.7 ± 0.8
**Total cholesterol (mg/dL)**	**185 ± 41**	**171 ± 34 ^2^**
HDL (mg/dL)	45 ± 13	45 ± 13
**Triglycerides (mg/dL)**	**194 ± 148**	**161 ± 70 ^3^**
LDL (mg/dL)	105 ± 37	95 ± 30
RCP (mg/dL)	0.54 ± 0.87	0.48 ± 0.62
HCO3 (mEq/L)	22.7 ± 3.2	23.6 ± 2.6
PTH (ng/mL)	90.7 ± 75.4	97.2 ± 57.9
Urine natrium (mEq/day)	142 ± 59	145 ± 60
Epoetin (UI/week)	0 (0–18,000)	0 (0–18,000)
Furosemide (mg/day)	25 (0–350)	25 (0–250)
**Hypotensive** agents (dose/day)	1.7 (0–4)	1.5 (0–4.5)
Statins (dose/pts/day) *	0.4 (0–2)	0.5 (0–2)
Omega fatty acids (g/day) *	0 (0–4)	0 (0–3)
**TUN g/24 h**	**10.8 ± 3.4**	**9.3 ± 3.1 ^4^**
**PCR g/24 h**	**67.5 ± 2.1**	**58.4 ± 19.4 ^5^**
**nPCR g/kg/24 h**	**0.90 ± 0.30**	**0.77 ± 0.20 ^6^**

**^1^***p* = 0.003; **^2^** *p* = 0.001; **^3^** *p* = 0.03; **^4^** *p* = 0.0006; **^5^** *p* = 0.0001; **^6^** *p* = 0.007. * Median (minimum–maximum). In bold, significant values.

**Table 3:** Changes in uremic toxins. Values are presented as mean ± SD.

	Probiotics Group		Placebo Group	
	T2	T5	T2	T5
**Lp-PLA2 (nmol/mL/min)**	162.9 ± 53.5	162.7 ± 37.9	**155.4 ± 39.3**	**167.5 ± 51.4 ^1^**
t-PC (μM)	124.1 ± 63.1	114.8 ± 56.6	116.7 ± 77.0	125.9 ± 90.0
f-PC (μM)	3.89 ± 2.13	4.52 ± 2.62	4.49 ± 3.86	5.38 ± 5.89
**t-IS (** **μ** **M)**	30.3 ± 23.7	28.1 ± 11.7	**30.1 ± 17.6**	**34.5 ± 20.2 ^2^**
f-IS (μM)	1.32 ± 1.07	1.29 ± 0.55	1.38 ± 0.92	1.55 ± 0.94

**^1^***p* = 0.006 T5 vs. T2 in the placebo group; **^2^** *p* = 0.026 T5 vs. T2 in the placebo group. In bold, significant values.

**Table 4:** Changes in biochemical parameters. Values are presented as mean ± SD.

	Probiotics Group		Placebo Group	
	T2	T5	T2	T5
eGFR (CKD-EPI, mL/min)	18.8 ± 5.1	19.9 ± 5.4	17.6 ± 4.3	17.3 ± 6.4
Mean creatinine and urea clearance (mL/min)	17.6 ± 5.7	17.9 ± 6.7	17.6 ± 5.3	18.7 ± 7.9
**Urine proteins (g/24 h)**	**1.2 ± 1.6** *	1.2 ± 1.7	**1.9 ± 2.1**	**2.7 ± 2.4 ^1^**
Hb (g/dL)	11.7 ± 1.5	12.0 ± 1.6	12.0 ± 1.5	12.2 ± 1.6
BUN (mg/dL)	47 ± 16	45 ± 16	46 ± 14	48 ± 18
Uric acid (mg/dL)	6.1 ± 1.4	5.7 ± 1.6	5.9 ± 1.0	5.9 ± 1.2
Albumin (mg/dL)	4.2 ± 0.3	4.1 ± 0.3	4.1 ± 0.3	4.1 ± 0.4
Calcium (mg/dL)	9.1 ± 0.6	8.9 ± 0.6	9.1 ± 0.4	9.1 ± 0.6
Phosphorus (mg/dL)	3.7 ± 0.9	3.6 ± 0.8	3.7 ± 0.7	3.9 ± 0.9
**Total cholesterol (mg/dL)**	174 ± 34	179 ± 31	**169 ± 36**	**185 ± 40 ^2^**
HDL (mg/dL)	43.9 ± 12.7	45 ± 11.4	47 ± 14	47 ± 12
Triglycerides (mg/dL)	161 ± 75	158 ± 70	162 ± 66	178 ± 92
**LDL (mg/dL)**	99 ± 31	102 ± 31	**90 ± 28**	**104 ± 35 ^3^**
RCP (mg/dL)	0.54 ± 0.71	0.48 ± 0.62	0.37 ± 0.47	0.6 ± 0.9
HCO3 (mEq/L)	24.1 ± 2.2	23.6 ± 2.9	23.1 ± 2.9	23.4 ± 3.0
PTH (ng/mL)	89.1 ± 48.8	81.0 ± 38.8	106.2 ± 65.7	120.6 ± 101.1
Urine natrium (mEq/day)	133 ± 62	135 ± 54	145 ± 60	162 ± 67
TUN g/24 h	9.1 ± 2.9	8.6 ± 3.5	9.5 ± 3.3	9.4 ± 3.6
PCR g/24 h	57.2 ± 1.8	56.0 ± 1.9	59.7 ± 2.9	61.6 ± 2.9
nPCR g/kg/24 h	0.74 ± 0.19	0.76 ± 0.18	0.81 ± 0.21	0.87 ± 0.31

**^1^***p* = 0.04 T5 vs. T2 in the placebo group; **^2^** *p* = 0.01 T5 vs. T2 in the placebo group; **^3^** *p* = 0.02 T5 vs. T2 in -placebo group; * *p* = 0.03 T2 in probiotics vs. placebo group. In bold, significant values.

**Table 5:** Changes in pharmacological therapy. Values are presented as median (minimum–maximum).

	Probiotics Group		Placebo Group	
	T2	T5	T2	T5
Epoetin (UI/week)	0 (0–8000)	0 (0–8000)	0 (0–8000)	0 (0–8000)
**Furosemide (mg/day)**	**25 (0–500)**	**0 (0–100) ^1^**	0 (0–125)	0 (0–125)
**Antihypertensive agents (dose/day)**	**1.5 (0–4.5)**	**1 (0–3) ^2^**	1 (0–4)	1 (0–4)
Statins (dose/pts/day)	0 (0–2)	0 (0–2)	0.5 (0–2)	0 (0–2)
Omega fatty acids (g/day)	0 (0–2)	0 (0–2)	0 (0–3)	0 (0–2)

**^1^***p* = 0.04 T5 vs. T2 in the probiotics group; **^2^** *p* = 0.008 T5 vs. T2 in the probiotics group. In bold, significant values.

**Table 6:** Nutritional assessment and quality of life (SF36 items). Values are presented as mean ± SD.

	Probiotics Group		Placebo Group	
	T2	T5	T2	T5
BMI (kg/cm^2^)	30.6 ± 10.2	30.6 ± 11.2	28.0 ± 5.9	28.0 ± 6.2
Free fat mass (kg)	52.8 ± 8.9	52.2 ± 8.9	52.6 ± 13.9	55.3 ± 9.8
Fat mass (kg)	24.8 ± 6.7	23.8 ± 6.9	21.6 ± 10.3	22.1 ±9.1
Angle phase	4.87 ± 0.90	4.95 ± 0.96	4.73 ± 1.3	5.1 ± 0.8
Hand grip (kg)	33.4 ± 11.7	30.7 ± 11.2	36.1 ± 8.5	34.6 ± 9.2
Physical functioning (points)	70.5 ± 20.2	66.5 ± 24.4	72.5 ± 23.3	72.8 ± 22.9
Physical role functioning (points)	41.4 ± 43.4	59 ± 39.8	60.3 ± 39.2	64.1 ± 41.1
Bodily pain (points)	62.7 ± 28.0	71.2 ± 27.7	73.6 ± 26.2	73.1 ± 29.9
General health perception (points)	46.3 ± 19.2	44.9 ± 20.3	53.2 ± 22.5	55.4 ± 19.1
Vitality (points)	48.1 ± 22.4	48.5 ± 24.9	56.9 ± 22.8	55.4 ± 26.3
Social role functioning (points)	69.8 ± 29.1	65.1 ± 30.7	73.1 ± 23.5	73.2 ± 6.7
**Emotional role functioning (points)**	**48.1 ± 42.8**	**57.7 ± 40.4 ^1^**	71.2 ± 41.5	70.8 ± 36.7
Mental health (points)	62.4 ± 22.3	64.5 ± 26.7	69.1 ± 18.2	66.9 ± 20.2

**^1^***p* = 0.04 T5 vs. T2 in the probiotics group. In bold, significant values.

## Data Availability

The data presented in the study are available on request from the corresponding author.
